# Pediatric Trauma in the Emergency Department: Clinical Risk Stratification, CT Utilization and Radiation Burden in a Tertiary Care Cohort

**DOI:** 10.3390/jcm15041470

**Published:** 2026-02-13

**Authors:** Mustafa Safa Pepele, Serdar Derya, Mahmut Murat, Adem Akdemir, Neslihan Yücel

**Affiliations:** Department of Emergency Medicine, Faculty of Medicine, İnönü University, Malatya 44069, Türkiye; serdar.derya@inonu.edu.tr (S.D.); mahmut.murat@inonu.edu.tr (M.M.); adem.akdemir@inonu.edu.tr (A.A.); neslihan.yucel@inonu.edu.tr (N.Y.)

**Keywords:** pediatric trauma, computed tomography, radiation dose, emergency department, risk stratification

## Abstract

**Background/Objective**: Pediatric trauma frequently prompts computed tomography (CT) in emergency departments; however, the cumulative radiation burden and its distribution across initial clinical risk strata remain incompletely characterized. We aimed to describe CT utilization and cumulative effective dose in a tertiary care pediatric trauma cohort and examine how radiation exposure accrues across pragmatic presentation-based risk groups. **Methods**: We conducted a retrospective cohort audit of pediatric trauma presentations at our institution. Risk stratification was based on the triage category and readily available initial physiological parameters. CT utilization and radiation burden were assessed at the patient level using the cumulative effective dose (mSv) derived from scanner dose metrics and region-specific conversion coefficients. Secondary analyses examined the dose distribution according to ED disposition and consultation pathways. Sensitivity analyses were performed using green triage only as an “ultra-low-risk” definition. **Results**: Among the 935 children, 545 (58.3%) underwent at least one CT examination. Although higher-risk categories had higher CT use and higher per-patient dose, a substantial share of the cohort’s cumulative radiation burden accrued in children initially classified as low-risk and/or ultimately discharged. Combined-region CT protocols contributed disproportionately to the higher dose categories. The findings were consistent in sensitivity analyses using a stricter ultra-low-risk definition. **Conclusions**: In this single-center audit, CT utilization and cumulative radiation burden were high, and non-trivial radiation exposure accrued among children initially classified as low-risk. These findings support targeted radiation stewardship interventions, including pathway optimization and the implementation of validated decision tools, where feasible, particularly for discharge-eligible and low-risk presentations.

## 1. Introduction

Trauma is one of the leading causes of morbidity and mortality in childhood, and injured children frequently present to emergency departments (EDs) for initial evaluation [1,2]. Although most pediatric trauma encounters are ultimately minor, clinicians must rapidly distinguish the small subset of children with clinically important injuries from the much larger group with self-limited conditions. Over the past two decades, computed tomography (CT) has become a cornerstone of trauma assessment, particularly for head, thoracic and abdominal injuries [3]. This widespread availability has improved the detection of occult injuries but has also raised concerns regarding potential overuse, especially in physiologically stable children.

Children are more susceptible to the harmful effects of ionizing radiation than are adults. Their tissues are more radiosensitive, and they have a longer remaining life expectancy in which radiation-induced malignancies may manifest [4,5,6,7,8,9,10]. Even though the absolute risk from a single CT scan is small for an individual child, the cumulative impact at the population level, and in children who undergo repeated imaging, is not trivial [4,5,6,7,8,9,10]. Consequently, pediatric societies and radiology organizations consistently emphasize the principles of justification and optimization: CT should be used only when it is expected to change management and, when used, should deliver the lowest dose reasonably achievable [4,5,6,7].

Several decision rules and practice guidelines have been developed to help clinicians safely reduce the use of CT in traumatized children. The PECARN head trauma rules, for example, identify children at very low risk of clinically important traumatic brain injury who can be managed without CT [11]. Similar efforts have been made for blunt head trauma using the CATCH and CHALICE rules, as well as more recent simplified decision tools for pediatric head injury and studies of CT yield in neurologically normal young children [12,13,14,15]. These tools, combined with careful clinical observation, have demonstrated that a substantial proportion of CT scans can be avoided without increasing missed injury rates [11,12,13,14,15]. Nevertheless, real-world data suggest that CT use remains high in many settings, and that a considerable proportion of pediatric trauma patients undergo advanced imaging despite relatively low clinical severity [3,15,16,17,18].

Most prior studies have focused on the proportion of children undergoing CT and on the diagnostic yield of these examinations, with less emphasis on how radiation exposure is distributed across different risk strata [3,9,16,17,18]. In routine practice, many pediatric trauma patients appear well at presentation; they are hemodynamically stable, have normal mental status and oxygenation, and are ultimately discharged home. If a meaningful fraction of the radiation burden is delivered to such clinically low-risk children, it would represent an important opportunity for quality improvement. Simultaneously, it is essential to ensure that efforts to reduce CT do not compromise care in truly high-risk patients for whom rapid cross-sectional imaging may be lifesaving.

Another underexplored aspect is the role of different surgical specialties in shaping imaging patterns. In many hospitals, pediatric trauma care involves orthopedics, pediatric surgery, and neurosurgery, each with its own threshold for requesting CT. Extremity injuries seen by orthopedics may require relatively low-dose imaging, whereas suspected head, thoracic or abdominal injuries in children evaluated by neurosurgery or pediatric surgery often lead to higher-dose head or body CT protocols [16,17]. Understanding how CT utilization and effective dose vary across these pathways can help tailor radiation-sparing strategies to areas with the greatest impact.

In this context, we aimed to describe CT utilization and cumulative radiation burden in pediatric trauma presentations in a tertiary care emergency department and examine how radiation exposure is distributed across initial clinical risk strata.

We hypothesized that, although higher-risk children would undergo more and higher-dose CT examinations, a substantial proportion of the cumulative radiation burden would still be delivered to clinically low-risk children who appear well at presentation and are often discharged. By characterizing this “radiation–risk mismatch,” we aimed to identify concrete targets for improving imaging practices in pediatric trauma without compromising the detection of clinically important injuries.

## 2. Materials and Methods

Study design and setting: this study was conducted in accordance with the STROBE statement.

We conducted a retrospective observational cohort study in the pediatric section of the Emergency Department (ED) of İnönü University Faculty of Medicine Hospital, a tertiary care referral center in Malatya, Türkiye. The hospital provides 24/7 pediatric emergency and surgical services and serves as a regional trauma-referral center.

Analytical approach: We evaluated CT utilization and radiation burden at both the patient level (cumulative effective dose per visit) and examination level (dose per CT), stratified by triage category and presentation physiology. We also examined radiation distribution among children who were ultimately discharged and across consultation pathways (orthopedics, pediatric surgery, and neurosurgery) as secondary analyses to identify potentially modifiable practice patterns relevant to radiation stewardship.

### 2.1. Study Population

The source population comprised all pediatric patients (aged 0–18 years) who presented to the ED with trauma-related complaints during the study period. For this analysis, we included consecutive patients who met the following criteria:

1. aged 0–18 years at the time of presentation;
2. ED visits for blunt or penetrating trauma;
3. triage category (green/yellow/red) assigned at ED registration;
4. at least one documented set of vital signs on arrival; and
5. a clearly recorded ED disposition (discharge, ward admission, or intensive care unit [ICU] admission).

Patients who met any of the following criteria were excluded:

1. left the ED against medical advice or before being evaluated by a physician;
2. were directly transferred to another facility without definitive assessment;
3. had missing key variables required for risk stratification, CT utilization, dose estimation, or ED disposition;
4. represented repeat visits for the same injury within the study period (only the index visit was retained).

During the study period, 1648 pediatric trauma presentations were screened for eligibility. After excluding cases with missing key variables required for risk stratification, CT utilization, dose estimation, or ED disposition (*n* = 521), 1127 presentations remained. We then excluded patients with burn injuries (*n* = 192) a priori because their imaging pathways and radiation exposure profiles differed substantially from those of blunt/penetrating trauma, yielding a final analytic cohort of 935 patients.

### 2.2. Triage Category and Mechanism of Injury

Triage was performed by trained ED nurses using a three-level, color-coded system and recorded as green (non-urgent), yellow (urgent), and red (emergent).

For descriptive analyses, triage was treated as a three-level categorical variable. In selected analyses related to the primary hypothesis, triage contributed to the definition of clinical risk groups (low vs. higher risk; see Section 2.6)

Validated pediatric decision rules (e.g., PECARN) could not be reconstructed retrospectively because several required elements (e.g., detailed symptoms, mechanism descriptors, and examination findings) were not consistently recorded in the structured fields. Therefore, risk grouping in this study was designed as a pragmatic clinical audit framework, rather than decision-rule validation.

The mechanism of injury was extracted from structured fields in the ED record and categorized as motor vehicle crash (MVC; including vehicle occupants and pedestrians), simple fall from standing height, fall from height, cut/laceration, and “other” mechanisms. In accordance with previous literature and clinical practice, high-energy mechanisms were defined as MVC and falls from a height.

### 2.3. Vital Signs and Clinical Assessments

The first set of vital signs obtained after ED arrival was extracted from the triage module of the electronic medical record as the earliest time-stamped measurements of heart rate and peripheral oxygen saturation (SpO_2_). When multiple sets were documented during the initial evaluation, we retained the earliest non-missing values to reflect the patients’ initial clinical status. We performed range and plausibility checks and treated implausible entries as missing data.

For descriptive purposes, vital signs were summarized as continuous variables and, given their age dependence, as categorical indicators of abnormal physiology using pre-specified pediatric reference values. These variables were used to characterize the overall cohort and support the definition of the clinical risk groups.

### 2.4. Specialty Consultations

We recorded whether the patient was evaluated by an orthopedist, pediatric surgeon, or neurosurgeon during the ED visit. These specialties were selected a priori because they represent the main surgical stakeholders in pediatric trauma at our institution and are likely to influence both imaging decisions and hospital admission. Consultation variables were coded as binary indicators (yes/no for each specialty), and patients could receive more than one consultation during the same encounter.

### 2.5. Imaging and Radiation-Dose Estimation

All computed tomography (CT) examinations performed during the ED visit were identified using a radiology information system. For each patient, we recorded whether CT was performed (yes/no) and, when available, the technical parameters for each examination, including the scanned body region (e.g., head, chest, abdomen/pelvis, or combined protocols), scan date and time, volumetric CT dose index (CTDI_vol, mGy), and dose–length product (DLP, mGy·cm), as reported on the scanner dose report.

The effective dose (E, mSv) for each CT examination was estimated by multiplying the DLP by age- and region-specific conversion coefficients (k, mSv/mGy·cm) derived from published pediatric dosimetry data based on the ICRP 103–compatible models. For patients who underwent more than one CT examination during the same ED visit, we calculated the per-patient cumulative effective dose as the sum of the effective dose values for all CT scans.

Conversion coefficients (k) were applied according to the age stratum and scanned body region; the full coefficient set and supporting references are provided in Supplementary Table S1.

To facilitate interpretation and reflect non-linear risk perception, we categorized the effective dose per patient into four pre-specified groups:

1. <1 mSv,
2. 1–< 3 mSv,
3. 3–<10 mSv, and
4. ≥10 mSv.

These thresholds were chosen to distinguish between very low-dose exposures, low-to-moderate diagnostic exposures, and higher-dose examinations that may be of greater concern for children.

### 2.6. Definition of Clinical Risk Groups

Because our primary hypothesis focused on the alignment between imaging-related radiation exposure and initial clinical severity, we pre-specified two risk definitions. For the primary analysis, patients were classified as presentation-based low-risk if they met all of the following criteria at presentation: (1) green or yellow triage category, (2) normal mental status (GCS = 15), and (3) preserved oxygenation (SpO_2_ ≥ 94%). The remaining patients were classified as high-risk. In sensitivity analyses, we defined discharged low-risk patients using the same criteria plus ED disposition of discharge (no ward or ICU admission).

### 2.7. Outcomes

CT yield (positive vs. negative) was determined by manually reviewing the official CT radiology reports for all patients who underwent CT during their ED visit. Examinations were coded as positive if the report documented any acute traumatic pathology (e.g., hemorrhage, fracture, organ injury, or pneumothorax/contusion) and negative if the report explicitly stated no acute traumatic findings. This yield was reported to contextualize imaging utilization and radiation burden; it should not be interpreted as adjudicated diagnostic accuracy for clinically important injuries.

The primary imaging outcomes were as follows.

1. the proportion of patients undergoing CT during the ED visit, and
2. the per-patient cumulative effective dose (mSv) in CT-exposed children.

The primary clinical outcome was hospital admission, defined as any ward or intensive care unit (ICU) admission to the ED. ED discharge without admission was considered “no admission.” ICU admission was also examined descriptively as a higher-severity outcome, but the small number of ICU admissions precluded the use of a robust multivariable model.

For the primary hypothesis (H1), we focused on the following aspects:

1. differences in CT utilization and effective dose between low- and higher-risk groups,
2. the distribution of the cumulative radiation burden (sum of effective doses) across these risk groups.

In secondary analyses, we evaluated CT utilization and effective dose according to specialty consultation (orthopedics, pediatric surgery, and neurosurgery) and explored the factors associated with hospital admission.

Statistical analysis: Continuous variables are reported as median [IQR] and compared using the Mann–Whitney U test. Categorical variables are reported as *n* (%) and were compared using the χ^2^ or Fisher’s exact test, as appropriate. Proportions are presented with 95% confidence intervals (CIs) using the Wilson method. For selected binary outcomes, risk ratios (RRs) with 95% confidence intervals were calculated using a log scale. Among the CT-imaged patients, 5/545 (0.9%) lacked DLP/E data and were excluded from the dose summaries; CT yield classification was unclear in 10/545 (1.8%) due to non-specific diagnostic entries. CT yield derived from the diagnosis field (TANI) was used as a proxy outcome. Sensitivity analysis: To test the robustness of potential misclassification, we repeated the key CT utilization and dose-distribution summaries using a stricter “ultra-low-risk” definition based on green triage only (regardless of other physiologic variables). Statistical analyses were performed using SPSS 22 for Windows (SPSS, Inc., Chicago, IL, USA).

Artificial Intelligence (AI) Use Statement: During the preparation of this manuscript, the authors used OpenAI ChatGPT (GPT-5.2 Thinking; OpenAI, San Francisco, CA, USA; accessed 11 February 2026) to assist with the language editing, clarity, and consistency of the written text and to help format sections according to journal requirements. No AI tool was used for the study design, data collection, statistical analysis, interpretation of results, or generation/alteration of original clinical data. All outputs were reviewed and edited by the authors, who took full responsibility for the content of the manuscript.

All statistical analyses were performed using commercially available software packages. Continuous variables were assessed for normality using visual inspection of histograms and Q–Q plots, together with the Shapiro–Wilk test. Normally distributed variables are reported as mean ± standard deviation (SD), whereas non-normally distributed variables are reported as median with interquartile range (IQR). Categorical variables are presented as counts and percentages.

Baseline characteristics were compared between children with and without hospital admission, clinical risk groups (low vs. high risk), and consultation categories. For group comparisons, we used Student’s *t*-test or Mann–Whitney U test for continuous variables, as appropriate, and the χ^2^ test or Fisher’s exact test for categorical variables.

To address the primary hypothesis, we compared the following:

CT utilization rates between the low- and high-risk groups and effective dose distributions (continuous and categorical) in the CT-exposed patients in each group.

We then calculated the cumulative effective dose in each risk group and expressed it as a proportion of the total radiation burden in the entire cohort exposed to CT scans.

In secondary analyses, we explored the association between selected clinical variables (age, sex, triage category, mechanism of injury, vital signs, and specialty consultations) and hospital admission using univariable logistic regression. Variables with *p* < 0.10 in the univariable analyses and those considered clinically relevant were included in a multivariable logistic regression model, provided that the number of outcome events allowed for adequate stability of the model.

All statistical tests were two-sided, and a *p*-value of <0.05 was considered statistically significant.

## 3. Results

### 3.1. Study Population and Baseline Characteristics

**Figure 1:**
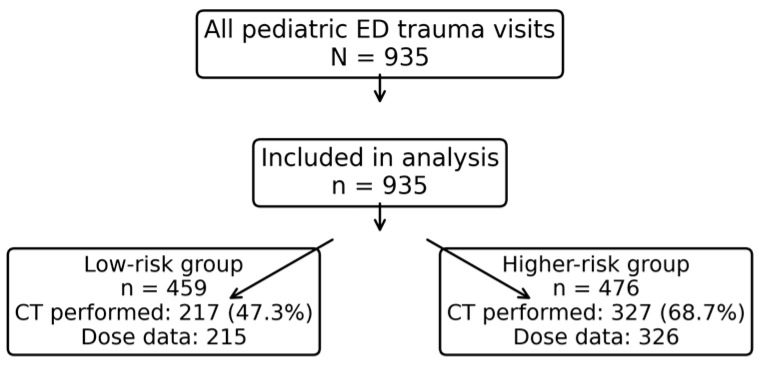
Flow diagram of patient inclusion and clinical risk group classification.

A total of 1648 pediatric trauma presentations were screened; 521 were excluded due to missing key variables, and 192 burn-injury cases were excluded, leaving 935 patients for the analysis. During the study period, 935 pediatric patients presented to the emergency department with trauma and met the inclusion criteria (Figure 1). The mean age was 8.9 ± 4.6 years (median 9 [interquartile range (IQR) 5–13] years), and 609 (65.1%) were male. Most children were triaged as yellow (urgent) (706/935, 75.5%), followed by green (non-urgent) (135/935, 14.4%) and red (emergent) (94/935, 10.1%). Simple falls from a standing height were the most common mechanism of injury (380/935, 40.6%), followed by motor vehicle crashes (200/935, 21.4%), falls from a height (75/935, 8.0%), cuts/lacerations (119/935, 12.7%), and other mechanisms (160/935, 17.1%).

On arrival, the patient’s vital signs were largely within normal limits. The median heart rate was 80 [78–85] beats/min and peripheral oxygen saturation was 97% [96–98%]. Only a small minority had markedly abnormal physiology, including tachycardia, bradycardia, hypoxia, or depressed mental status (GCS < 15 in 3.0% and GCS ≤ 8 in 1.9% of patients). Orthopedics was the most frequently involved specialty (698/935, 74.7%), followed by pediatric surgery (318/935, 34.0%) and neurosurgery (157/935, 16.8%). Overall, 545/935 (58.3%) children underwent at least one CT examination, 473/935 (50.2%) were admitted to the hospital (ward or ICU), and 462 (49.8%) were subsequently discharged. Seven-day revisits occurred in 46 (4.9%) patients, 30-day revisits in 22 (2.4%) patients, and in-hospital mortality in 9 (1.0%) patients.

The baseline characteristics according to the admission status are summarized in Table 1. Compared with the discharged children, those who were admitted were slightly older (median 9 [6–14] vs. 8.5 [5–12] years; *p* = 0.004), more often male (72.3% vs. 57.9%; *p* < 0.001), more frequently triaged as yellow or red, and more likely to have high-energy mechanisms (motor vehicle crashes and falls from height) and undergo CT (69.1% vs. 47.2%; all *p* < 0.001).

### 3.2. Clinical Risk Groups and CT Utilization

Figure 1 Study flow diagram. Of the 1648 pediatric trauma presentations screened, 521 were excluded due to missing key variables. Of the 1127 eligible presentations, 192 burn-injury cases were excluded, yielding a final cohort of 935 patients.

Overall, of 545/935 children (58.3%) underwent at least one CT examination. CT utilization differed markedly among the risk strata (Table 2). CT was performed in 451/834 low-risk children (54.1%; 95% CI, 50.7–57.4) and 94/101 higher-risk children (93.1%; 95% CI 86.4–96.6).

Sensitivity analysis (ultra-low-risk = green triage only): Among Green-triage children (*n* = 135), 52/135 underwent CT (38.5%), compared with 492/800 (61.5%) among yellow/red triage. Despite a lower per-patient dose in green triage CT recipients (median 0.055 mSv vs. 2.50 mSv), green triage presentations accounted for 1.9% of the cohort’s total cumulative effective dose.

**Figure 2:**
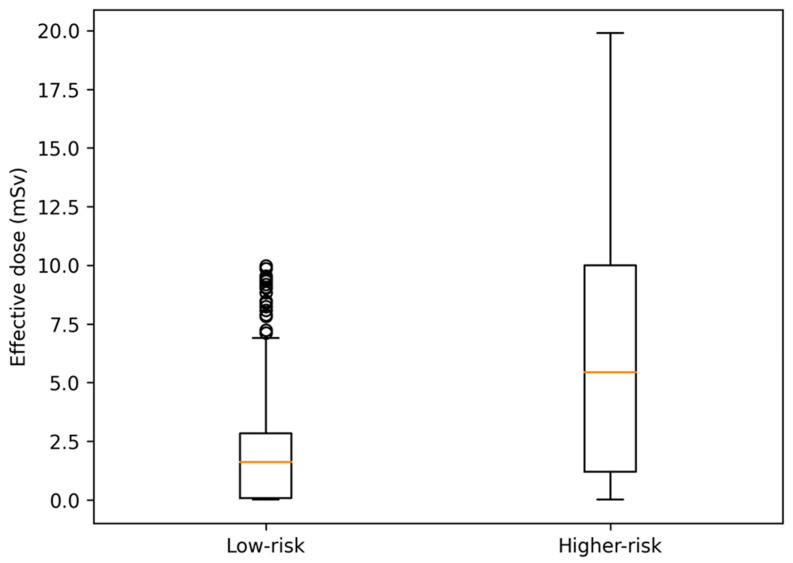
Distribution of effective dose among CT-exposed children by clinical risk group. The central line indicates the median; the box represents the interquartile range (IQR); whiskers indicate 1.5 × IQR; circles represent outliers.

The median cumulative effective dose (E) was 1.55 [0.03–6.55] mSv overall, 0.36 [0.03–4.50] mSv in low-risk CT recipients, and 8.25 [4.00–16.38] mSv in higher-risk CT recipients. E ≥ 10 mSv occurred in 50/446 low-risk (11.2%; 95% CI 8.6–14.5) versus 40/94 higher-risk CT recipients (42.6%; 95% CI 33.0–52.6), RR 0.26 (95% CI 0.19–0.37). CT yield and cumulative radiation burden by risk group are summarized in Table 3, and region-specific CT yield is shown in Table 4. The distribution of the effective dose categories by risk group is shown in Figure 2.

### 3.3. Radiation Dose Distribution by Clinical Risk Group

Dose information was available for the of 540/545 CT examinations (99.1%). The median effective dose was 1.55 [0.03–6.55] mSv overall, 0.36 [0.03–4.50] mSv in low-risk CT recipients, and 8.25 [4.00–16.38] mSv in higher-risk CT recipients; E ≥ 10 mSv occurred in 50/446 low-risk versus 40/94 higher-risk CT recipients (RR 0.26, 95% CI 0.19–0.37). Among the CT-exposed patients with dose information (*n* = 540), the median dose–length product (DLP) was 423.5 [107.0–988.8] mGy·cm, corresponding to a median cumulative effective dose of 1.55 [0.03–6.55] mSv (mean 4.93 ± 7.69 mSv; range 0.002–57.3 mSv). Overall, 303/540 (56.1%) examinations were <3 mSv, 147/540 (27.2%) were 3–<10 mSv, and 90/540 (16.7%) were ≥10 mSv.

**Figure 3:**
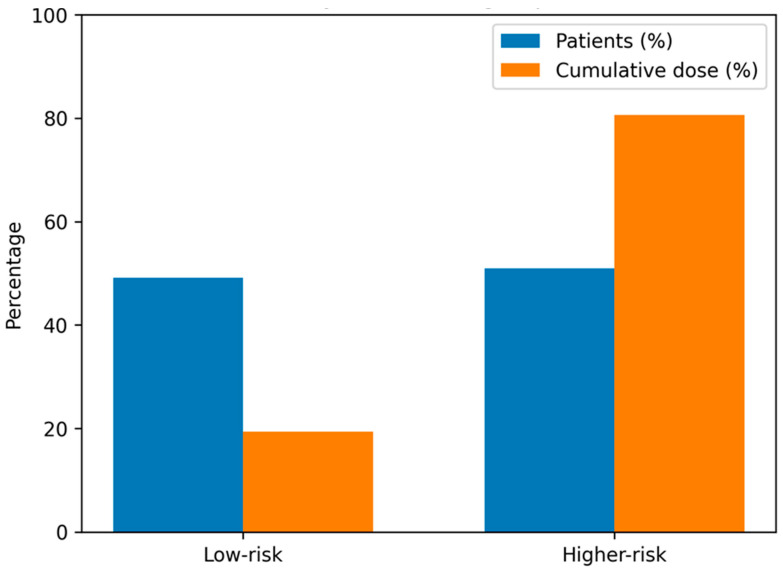
Proportion of patients and cumulative effective dose by clinical risk group.

When examining the cumulative radiation burden, the total effective dose delivered to all CT-exposed children with dose information was 2665.4 mSv. Of this, 1628.2 mSv (61.3%) accrued in the initial low-risk group and 1027.2 mSv (38.7%) in the higher-risk group, reflecting the much larger number of low-risk children undergoing CT, despite the higher per-patient doses in the higher-risk group. The distribution of the cumulative effective dose across the risk strata is illustrated in Figure 3.

### 3.4. CT Utilization and Dose by Specialty Consultation (Secondary Analysis)

CT utilization and per-patient effective dose also varied by consulting service, with higher CT use and higher cumulative effective dose among neurosurgical and pediatric surgical consultations (Supplementary Table S2).

### 3.5. CT Yield Based on Radiology Report Review

Radiology reports were available for 542 of the 544 children who underwent CT. Overall, 405/542 (74.7%) CT examinations revealed at least one acute traumatic pathology. Among children who met the low-risk criteria at presentation, 319/448 (71.2%) were coded as positive, compared with 86/94 (91.5%) in the higher-risk group. The low-risk group accounted for 61.3% of the cumulative doses. In the sensitivity analysis, the ultra-low-risk subgroup (Green triage, GCS = 15, SpO_2_ ≥ 94%) contributed 2.0% of the total effective dose.

## 4. Discussion

In this single-center cohort of 935 pediatric trauma patients, we found that CT was used in more than half of the children presenting to the emergency department and that nearly one in two patients was admitted to the hospital. As expected, higher-acuity presentations (older age, male sex, red triage, and high-energy mechanisms such as motor vehicle crashes and falls from height) were associated with increased CT utilization and a higher likelihood of hospital admission (Table 1). Despite this, most children arrived with stable vital signs, normal oxygen saturation, and a Glasgow Coma Scale (GCS) score of 15, underscoring that the majority of pediatric trauma consultations in our setting involve physiologically well-compensated patients.

CT utilization in our cohort (58.3%) was higher than that reported in many international pediatric trauma series, particularly those restricted to isolated minor head or abdominal trauma cases. Several context-specific factors may explain this: as a tertiary referral center, our ED receives interfacility transfers and higher-energy mechanisms; imaging decisions are often influenced by specialty consultations and the need for rapid disposition; and local practice patterns and medicolegal risk tolerance may favor CT over prolonged observation, particularly outside daytime hours when alternative modalities (e.g., expert ultrasonography or MRI) may be less readily available. Importantly, we present these findings as an institutional audit rather than a benchmark; the high utilization should be interpreted as a stewardship target and quality-improvement opportunity, especially for low-risk and discharge-eligible children and combined-region CT protocols.

Notably, CT positivity remained substantial even among children who met our pragmatic ‘low-risk’ criteria (71.2%). In our setting, the yellow triage category often reflects moderate acuity without clear physiologic compromise and was therefore grouped with green for this audit; however, triage and initial physiology do not incorporate mechanism-based decision rules. Therefore, the observed positivity should not be interpreted as evidence that physiologically low-risk children uniformly have high injury rates; rather, it likely reflects (i) selection for imaging—clinicians tend to order CT in a subset of stable children when mechanism, symptoms, or examination raise concern; (ii) the broad definition of positivity, which captured any acute traumatic pathology on radiology reports, including minor findings that may not meet thresholds for clinically important injury; and (iii) residual misclassification inherent to retrospective risk stratification. For transparency, we performed sensitivity analyses using a stricter ‘ultra-low-risk’ definition (green triage only), which yielded consistent qualitative conclusions regarding non-trivial radiation exposure in apparently low-risk presentations.

Limitations: This study had several limitations. First, this was a single-center retrospective cohort study; therefore, practice patterns (including CT thresholds and the use of multi-region scanning) may differ across systems and limit external generalizability. Second, our clinical risk stratification was intentionally pragmatic and based on routinely recorded ED variables (triage category, GCS, and oxygen saturation) rather than validated decision rules (e.g., PECARN), which were not reliably retrievable from medical records; misclassification remains possible. Third, CT yield was coded from radiology reports as the presence of any acute traumatic pathology; this broad definition does not distinguish ‘clinically important injuries’ and cannot attribute findings to a specific CT region in multi-region examinations. Finally, dose information was unavailable for a small minority of CT examinations, and we could not assess long-term outcomes or downstream imaging outside our institution.

Clinical implications: These findings support the use of risk-aligned imaging pathways for pediatric trauma. Practical strategies include embedding validated decision rules into ED workflows, standardizing consultation-driven imaging pathways, implementing dose tracking with audit-and-feedback, and prioritizing non-ionizing modalities when clinically appropriate.

When examining the cumulative radiation burden, the total effective dose across all CT examinations with dose data was 2655.4 mSv; 1628.2 mSv (61.3%) accrued in the initial low-risk group and 1027.2 mSv (38.7%) in the higher-risk group, respectively.

Our data also show that radiation exposure is unevenly distributed across different surgical specialties. While orthopedics managed the largest number of patients, CT utilization and per-patient effective dose were comparatively modest in this group, consistent with a predominance of extremity and low-dose regional imaging [16]. In contrast, children evaluated by pediatric surgery and neurosurgery almost uniformly underwent CT and received substantially higher doses, reflecting both the need for head, thoracoabdominal, or whole-body CT and the concentration of more severe injuries in these areas. From a systems perspective, this suggests that radiation-sparing strategies may need to be tailored differently for large-volume, lower-dose pathways (orthopedics) and smaller but high-dose pathways (pediatric and neurosurgery).

Our findings are broadly consistent with previous work showing increased CT use in pediatric trauma over the past two decades, often out of proportion to the rate of clinically important injuries [3,15,16,17,18]. Several studies have reported high CT utilization in children with minor head or torso trauma, despite low rates of neurosurgical intervention or intraabdominal injury, and have highlighted substantial variability between institutions and clinicians [3,15,16,17,18]. Clinical decision rules such as PECARN, CATCH and CHALICE, as well as more recent simplified rules and observational data on CT yield in neurologically normal children, have demonstrated that a considerable fraction of CT scans can be safely avoided in carefully selected low-risk children [11,12,13,14,15,17]. Our results extend this literature by quantifying the overall radiation burden delivered to children who appear to be low-risk according to simple, routinely available ED variables and who are ultimately discharged.

The dose levels observed in our cohort were comparable to those reported in contemporary pediatric imaging and trauma studies. The median effective dose for head and torso CT is typically in the range of 2–7 mSv, with higher values for multiphase or whole-body protocols [4,5,6,7,8,9,10]. In our study, almost half of the CT-exposed children received less than 1 mSv, but approximately one in six received ≥ 10 mSv. Given the increased lifetime susceptibility of children to radiation-related malignancies and the cumulative nature of the risk, even relatively small shifts in CT use in low-risk patients may have meaningful implications at the population level [4,5,6,7,8,9,10]. Simultaneously, for truly high-risk trauma, CT remains a cornerstone of timely diagnosis, and our data reassure that most of the radiation burden is concentrated in children with higher clinical acuity. Although individual-level cancer risk is not directly measurable in our dataset, these dose strata are clinically meaningful in pediatrics, where cumulative exposure and younger age at irradiation are associated with higher lifetime attributable risk, reinforcing the rationale for radiation stewardship, especially when the expected diagnostic benefit is low.

These findings have several implications for clinical practice and quality improvement programs. First, the fact that nearly one-fifth of the total effective dose occurred in low-risk discharged patients suggests that there is room to strengthen decision-making regarding imaging in this subgroup. Structured observation, greater use of clinical decision rules, and more systematic incorporation of mechanisms of injury and physical examination findings could reduce potentially avoidable CT in children who are well appearing and hemodynamically stable [11,12,13,14,15,17]. Second, targeted interventions might focus on high-yield “pressure points,” such as routine head CT in neurologically normal children with minor mechanisms of injury or abdominal CT in the absence of concerning examination findings or laboratory abnormalities. Third, multidisciplinary collaboration with radiology, pediatrics, and surgery is likely to be essential, as imaging pathways are shared across services, and any change must be safe and acceptable to all stakeholders.

Our study also underscores the importance of transparent dose monitoring at an institutional scale. The ability to estimate the per-patient and cumulative effective dose and link these data with clinical outcomes and consultation patterns provides a practical framework for local audits [4,5,6,7,8,9,10,16,17,18]. Regular feedback to clinicians about radiation exposure in different risk strata, combined with education on pediatric dosimetry and optimization techniques, may help align imaging practices more closely with clinical severity. In addition, embedding simple risk stratification tools into electronic medical records could facilitate the real-time identification of low-risk children in whom CT may be deferred or replaced by ultrasonography or serial examination.

This study had some limitations. First, it was conducted at a single tertiary care center, and the findings may not be generalizable to settings with different trauma systems, CT availability, or imaging protocols. Second, the retrospective design relies on the accuracy and completeness of electronic documentation; vital signs, in particular, may be affected by rounding or default entries, which could introduce misclassification of physiological derangements in the present study. Third, our clinical risk constructs were intentionally pragmatic and based on routinely available ED variables, which may not fully capture injury severity compared to formal trauma scores. Fourth, the effective dose was estimated from the scanner-reported DLP values using published conversion coefficients rather than direct organ dose measurements, which introduced uncertainty in the absolute dose estimates. Finally, severe outcomes were infrequent, limiting robust multivariable modeling for the most extreme endpoints.

Despite these limitations, this study had several notable strengths. We analyzed a relatively large, unselected cohort of pediatric trauma patients with detailed information on triage, vital signs, consultations, imaging use, and estimated radiation doses. Our risk group definition is simple and relies on routinely collected variables, making the approach easily reproducible in other EDs. By focusing not only on CT utilization rates but also on the distribution of cumulative effective dose across clinical risk strata, we provide a nuanced view of how radiation is allocated within the pediatric trauma population.

In conclusion, CT use in pediatric trauma at our institution is partly aligned with clinical severity, with higher-risk children receiving more imaging and higher doses. However, a substantial minority of the total radiation burden is still delivered to children who appear to be low-risk based on routine ED measures and are discharged home. Future studies should aim to validate these findings in other settings, integrate formal decision rules and dose monitoring into clinical workflows, and evaluate whether targeted interventions can safely reduce radiation exposure in low-risk pediatric trauma patients without compromising the detection of clinically important injuries.

## Figures and Tables

**Table 1:** Baseline characteristics and imaging/radiation metrics in pediatric trauma patients according to clinical risk group.

Variable	Overall (*n* = 935)	Discharged (*n* = 462)	Admitted (*n* = 473)	*p*
Age, years, median [IQR]	9.0 [5.0–13.0]	8.0 [5.0–12.0]	10.0 [6.0–14.0]	0.002
Male sex, n (%)	609 (65.1%)	267 (57.8%)	342 (72.3%)	<0.001
Mechanism of injury, n (%)	In-vehicle traffic (AITK): 64 (6.8%) Out-of-vehicle traffic (ADTK): 136 (14.5%) Simple fall: 380 (40.6%) Fall from height: 75 (8.0%) Laceration: 119 (12.7%) Other: 160 (17.1%) Unknown: 1 (0.1%)	In-vehicle traffic (AITK): 18 (3.9%) Out-of-vehicle traffic (ADTK): 33 (7.1%) Simple fall: 241 (52.2%) Fall from height: 26 (5.6%) Laceration: 45 (9.7%) Other: 99 (21.4%) Unknown: 0 (0.0%)	In-vehicle traffic (AITK): 46 (9.7%) Out-of-vehicle traffic (ADTK): 103 (21.8%) Simple fall: 139 (29.4%) Fall from height: 49 (10.4%) Laceration: 74 (15.6%) Other: 61 (12.9%) Unknown: 1 (0.2%)	<0.001
Triage color (green/yellow/red), n (%)	Green: 135 (14.4%) Yellow: 706 (75.5%) Red: 94 (10.1%) Unknown: 0 (0.0%)	Green: 90 (19.5%) Yellow: 369 (79.9%) Red: 3 (0.6%) Unknown: 0 (0.0%)	Green: 45 (9.5%) Yellow: 337 (71.2%) Red: 91 (19.2%) Unknown: 0 (0.0%)	<0.001
GCS = 15, n (%)	905 (96.8%)	460 (99.6%)	445 (94.1%)	<0.001
SpO2 at presentation, %, median [IQR]	97 [96–98]	97 [96–98]	97 [96–98]	0.598
Heart rate, bpm, median [IQR]	80 [78–85]	80 [77–85]	80 [79–89]	<0.001
Any specialty consultation, n (%)	935 (100.0%)	462 (100.0%)	473 (100.0%)	—
Any CT performed, n (%)	545 (58.3%)	215 (46.5%)	330 (69.8%)	<0.001
7-day revisit, n (%)	46 (4.9%)	26 (5.6%)	20 (4.2%)	0.323
30-day revisit, n (%)	22 (2.4%)	8 (1.7%)	14 (3.0%)	0.215
In-hospital mortality, n (%)	9 (1.0%)	1 (0.2%)	8 (1.7%)	0.038

**Table 2:** CT utilization and radiation burden by body region and clinical risk group.

Body Region/Protocol	CT *n* (%)			E (mSv), Median [IQR]			DLP Median [IQR]	*p*
	Overall	Low-Risk	Higher-Risk	Overall	Low-Risk	Higher-Risk	Overall	—
Head (non-contrast)	4 (0.4%)	4 (0.5%)	0 (0.0%)	1.20 [1.16–1.40]	1.20 [1.16–1.40]	—	387 [352–480]	—
Face/sinus	0 (0.0%)	0 (0.0%)	0 (0.0%)	—	—	—	—	—
Cervical spine	1 (0.1%)	1 (0.1%)	0 (0.0%)	1.40 [1.40–1.40]	1.40 [1.40–1.40]	—	349 [349–349]	—
Chest	6 (0.6%)	5 (0.6%)	1 (1.0%)	1.69 [0.79–2.38]	1.38 [0.60–2.50]	2.00 [2.00–2.00]	112 [52–162]	—
Abdomen-pelvis	16 (1.7%)	16 (1.9%)	0 (0.0%)	0.85 [0.50–3.17]	0.85 [0.50–3.17]	—	58 [28–198]	—
Combined regions (multi-area)	191 (20.4%)	136 (16.3%)	55 (54.5%)	5.50 [3.13–10.05]	5.05 [3.00–9.51]	7.50 [3.62–10.70]	836 [566–1393]	0.105
Whole-body CT (WBCT)	80 (8.6%)	44 (5.3%)	36 (35.6%)	9.10 [4.95–18.00]	7.55 [4.57–17.11]	13.05 [6.80–19.98]	1292 [815–1964]	0.083
Extremity/other	247 (26.4%)	245 (29.4%)	2 (2.0%)	0.03 [0.02–0.10]	0.03 [0.02–0.10]	0.09 [0.05–0.12]	111 [74–224]	0.923

**Table 3:** CT yield (radiology report-based) and cumulative radiation burden according to the clinical risk group.

Group	CT *n*	Positive *n*	Yield %	Cumulative ED (mSv)	Dose Share %
Low risk at presentation	448	319	71.2	1628.2	61.3
Higher risk at presentation	94	86	91.5	1027.2	38.7
Low-risk discharged	211	136	64.5	510.4	19.2
Ultra-low-risk (Green triage)	52	41	78.8	51.8	2.0

**Table 4:** CT yield (radiology report-based) among children receiving CT by body region and clinical risk group (multi-region scans overlap).

CT Region	CT *n* (Total)	Yield % (Total)	CT *n* (Low Risk)	Yield % (Low Risk)	CT *n* (Higher Risk)	Yield % (Higher Risk)
Abdomen	243	64.6	156	49.4	87	92.0
Chest	252	65.9	161	50.9	91	92.3
Extremity	398	81.4	341	79.8	57	91.2
Head (brain)	259	65.6	171	52.0	88	92.0
Maxillofacial	105	81.0	54	64.8	51	98.0
Pelvis	28	78.6	14	57.1	14	100.0
Spine	232	67.2	151	53.6	81	92.6

## Data Availability

The datasets used and/or analyzed in the current study are available from the corresponding author upon reasonable request.

## Supplementary Materials

The following supporting information can be downloaded at: https://www.mdpi.com/article/10.3390/jcm15041470/s1, Table S1: Age- and region-specific DLP-to-effective-dose conversion coefficients; Table S2: Consultation patterns and imaging/radiation metrics.

## Abbreviations

CT: computed tomography; DLP, dose-length product; ED, emergency department; ICU, intensive care unit; MVC, motor vehicle collision; mSv, millisievert.
