# Supplementary material for: Pediatric Trauma in the Emergency Department: Clinical Risk Stratification, CT Utilization and Radiation Burden in a Tertiary Care Cohort
Source: J Clin Med. 2026 Feb 13;15(4):1470. doi: 10.3390/jcm15041470 (PMC12941996; doi:10.3390/jcm15041470)
Supplement: Supplementary file 1 [file jcm-15-01470-s001.zip › jcm-4095679-supplementary.pdf]

Supplementary Materials

Table S1. Age- and region-specific DLP-to-effective-dose conversion coefficients (k).

Notes: Enter the conversion coefficient k (mSv/mGy·cm) used to convert the DLP to the effective dose for each age band and CT body region. If multiple published sources are used, specify the selected source in the final column. Values marked with * were estimated from small strata (n<5) and should be interpreted cautiously.

Table S1. Age- and region-specific DLP-to-effective-dose conversion coefficients (k).

**Age band**	**CT region**	**k (mSv/mGy·cm)**	**Reference / source**
0–<1 year	Head	0.00413*	Derived from study dataset (E/DLP); small-n
0–<1 year	Cervical spine	—	—
0–<1 year	Chest	—	—
0–<1 year	Abdomen–pelvis	—	—
0–<1 year	Whole-body / multi-region	0.00909	Derived from study dataset (E/DLP)
1–<5 years	Head	0.00349*	Derived from study dataset (E/DLP); small-n
1–<5 years	Cervical spine	0.00401*	Derived from study dataset (E/DLP); small-n
1–<5 years	Chest	—	—

1–<5 years	Abdomen–pelvis	0.01011*	Derived from study dataset (E/DLP); small-n
1–<5 years	Whole-body / multi-region	0.00490	Derived from study dataset (E/DLP)
5–<10 years	Head	0.00209*	Derived from study dataset (E/DLP); small-n
5–<10 years	Cervical spine	—	—
5–<10 years	Chest	0.01481*	Derived from study dataset (E/DLP); small-n
5–<10 years	Abdomen–pelvis	0.01477	Derived from study dataset (E/DLP)
5–<10 years	Whole-body / multi-region	0.00296	Derived from study dataset (E/DLP)
10–<15 years	Head	—	—
10–<15 years	Cervical spine	—	—
10–<15 years	Chest	0.01497*	Derived from study dataset (E/DLP); small-n
10–<15 years	Abdomen–pelvis	0.01518	Derived from study dataset (E/DLP)
10–<15 years	Whole-body / multi-region	0.00160	Derived from study dataset (E/DLP)

15–18 years	Head	—	—
15–18 years	Cervical spine	—	—
15–18 years	Chest	0.01570*	Derived from study dataset (E/DLP); small-n
15–18 years	Abdomen–pelvis	0.01536*	Derived from study dataset (E/DLP); small-n
15–18 years	Whole-body / multi-region	0.00485	Derived from study dataset (E/DLP)

Table S2. Consultation patterns and imaging/radiation metrics.

Notes: Population counts and radiation metrics were obtained by consulting the service. The DLP was reported in mGy·cm and effective dose as E (mSv), both as median [IQR].

Table S2. Consultation patterns and imaging/radiation metrics.

Consulting service	Consultations, n (%)	Any CT, n (%)	Total DLP, median [IQR]	Effective dose (E), median [IQR]	≥10 mSv, n (%)
Neurosurgery	157 (16.8%)	156 (99.4%)	1024 [638–1661]	6.85 [3.72–12.62]	56 (35.9%)

Pediatrics	322 (34.4%)	247 (76.7%)	903 [528–1542]	6.50 [3.29–11.70]	81 (32.8%)
Orthopedics	700 (74.9%)	380 (54.3%)	220 [88–786]	0.10 [0.02–4.00]	49 (12.9%)
General Surgery	0 (0.0%)	0 (0.0%)	—	—	0 (0.0%)
ENT	2 (0.2%)	2 (100.0%)	1948 [1571–2324]	11.07 [7.31–14.84]	1 (50.0%)
Ophthalmology	2 (0.2%)	2 (100.0%)	1766 [1299–2233]	11.16 [7.43–14.88]	1 (50.0%)
Other / multiple	188 (20.1%)	180 (95.7%)	1078 [638–1830]	7.15 [3.78–15.00]	68 (37.8%)
